# Formulation Development and Evaluation of the Therapeutic Efficacy of Brinzolamide Containing Nanoemulsions

**Published:** 2017

**Authors:** Mohammad Mehdi Mahboobian, Ali Seyfoddin, Ilva D. Rupenthal, Reza Aboofazeli, Seyed Mohsen Foroutan

**Affiliations:** a *Department of Pharmaceutics, School of Pharmacy & Protein Technology Research Center, Shahid Beheshti University of Medical Sciences, Tehran, Iran. *; b *School of Applied Sciences, Auckland University of Technology, New Zealand.*; c *Buchanan Ocular Therapeutics Unit, Department of Ophthalmology, New Zealand National Eye Centre, Faculty of Medical and Health Sciences, University of Auckland, New Zealand.*

**Keywords:** Ocular drug delivery, Brinzolamide, Nanoemulsion, Physicochemical characterization, Ocular bioavailability, Therapeutic efficacy

## Abstract

Brinzolamide (BZ) is an intraocular pressure reducing agent with low bioavailability. The purpose of the present study was to overcome this issue by development of BZ containing nanoemulsions (NEs) as an ocular drug delivery system with desirable therapeutic efficacy. Brinzolamide NEs were prepared by the spontaneous emulsification method. Based on initial release studies, twelve formulations with the slowest release characteristics were subjected to further physicochemical investigations such as particle size, polydispersity index, pH, refractive index, osmolality and viscosity. The therapeutic efficacy of these formulations was assessed by measuring the intraocular pressure after instillation of the prepared NEs in normotensive albino rabbit eyes. Nanoemulsions with suitable physicochemical properties exhibited high formulation stability under different conditions. more over biological evaluations indicated that using lower drug concentrations in NE formulations (0.4%) had a similar or even better pharmacodynamic effect compared to the commercial suspension with a higher drug concentration (1%). Our findings suggest that NEs could be effectively used as carriers for enhancing the bioavailability of topically applied ophthalmic drugs.

## Introduction

Topical application of drugs in the form of solutions is a popular route for the treatment of anterior segment eye diseases. Despite convenient manufacturing and easy administration, achieving desirable therapeutic effects in intraocular tissues remains a major problem (1). Various precorneal factors such as nasolacrimal drainage and blinking remove most of the instilled drug from the surface of the eye in less than 30 s (2). In addition, the multi-layered corneal barrier and tear film structure limit the penetration of drugs into the inner sections of the eye. The cornea consists of five main layers: epithelium, Bowman’s membrane, stroma, Descemet’s membrane and endothelium (3). Diffusion through the hydrophilic stroma is the rate limiting factor for permeation of water insoluble drugs while hydrophilic drugs have difficulty crossing the lipophilic epithelium (4). Based on these unique features and the fast nasolacrimal drainage from the ocular surface, the bioavailability of most topically instilled drugs generally remains less than 5% (5).

To overcome these limitations and improve the therapeutic efficacy of drugs applied to the ocular surface, different strategies such as viscosity enhancing agents (6), in situ-gelling systems (7), iontophoresis (8) and implants (9) have been investigated. In recent years, colloidal carriers such as liposomes, nanoparticles and nanoemulsions have also been investigated for ophthalmic drug delivery (10). Nanoemulsions (NEs) are colloidal carriers with nanoscopic droplets of less than 200 nm in size. These heterogeneous systems usually consist of oil droplets in an aqueous medium stabilised with a mixture of surfactant and co-surfactant (S_mix_) molecules (11). NEs offer many advantages such as high kinetic stability, ease of production and high solubilising capacity which render them attractive systems for drug delivery (12), with ocular drug delivery being one the most important applications of NEs. NE formulations have been investigated for enhancing ocular bioavailability (13, 14), increasing drug stability (15), reducing adverse effects (16) and providing sustained release of ophthalmic drugs (17, 18). 

Brinzolamide (BZ) is a water insoluble drug and is formulated commercially as a 1% aqueous suspension (Azopt^®^). BZ acts as an intraocular pressure (IOP) reducing agent by inhibiting carbonic anhydrase enzyme type II and is used as a first line medication for the treatment of open angle glaucoma in mono or combination therapy with beta-blockers and prostaglandins (19). The aqueous suspension of BZ has a low bioavailability due to the need for solubilisation of particles on the surface of the eye before the precorneal elimination processes occurs (20). Moreover, using a 1% suspension can cause blurred vision (21); however, using lower concentrations of BZ resulted in an insufficient therapeutic effect (22). To overcome these types of limitations, other carbonic anhydrase inhibitors like dorzolamide and acetazolamide have been formulated into NEs to efficiently deliver the drug to the ocular tissues and maintain therapeutic efficacy over prolonged periods 

(17, 18).

The aim of this work was to develop NEs with lower concentrations of BZ (0.4%) to reduce drug adverse effects and achieve higher bioavailability and to evaluate their *in-vivo* therapeutic efficacy by measuring the IOP in normotensive New Zealand albino rabbits.

## Experimental

*Materials*

BZ was a gift from Bachem (Switzerland). Brij 35 and Tyloxapol were purchased from Sigma (USA). Triacetin was supplied from Merck Chemical Co. (Germany). Cremophore RH40 was gifted by Osve Pharmaceutical Co. (Iran). Labrasol, Transcutol P and Capryol90 were provided as gift samples by Gattefosse (France). Purified water was obtained from MilliQ water purification systems (Millipore, France). All other chemicals used were of analytical grade.

*Development of BZ NEs*

The spontaneous emulsification method was used for the preparation of BZ oil-in-water (O/W) NEs. In this method appropriate amounts of oil, surfactant and co-surfactant were mixed together and BZ (0.4% w/w) was then incorporated into this mixture. After obtaining a homogenous mixture, water was added to the system under continuous stirring for 1 h. In each formulation, the amount of oil and surfactant to co-surfactant mixture (S_mix_) was kept constant at 5% and 20% w/w respectively. For the development of different secondary formulations, the amount of oil and S_mix_ increased up to 1.5 fold compared to each primary formulation (Table 1).

*In-vitro drug release studies*

The dialysis bag method was used to study the release behaviour of BZ containing NEs. Initially, 1 ml of each formulation was poured into a cellulose dialysis bag (MW cut-off 12,400 Da) that was previously soaked in distilled water at 4 ºC for 24 h. The dialysis bag was connected to the paddle of a USP apparatus II with 50 rpm rotation in 250 mL release medium consisting of phosphate buffer (pH=7.4). The temperature was set to 34.0 ± 0.2 ^º^C similar to the surface temperature of the eye (23).

Release studies were carried out for 6 h under sink conditions and at defined intervals (5, 10, 20, 30, 60, 120, 180, 240, 300 and 360 min), 4 mL of medium was taken out and replaced with the same volume of fresh medium. The amount of BZ released from NEs at each time interval was measured with UV spectrophotometry at 254 nm and the percentage of release efficiency (RE) was calculated using the following formula (24):

$\mathrm{RE}=\frac{\int_{0}^{t}y\times \mathrm{dt}}{\mathrm{y100}\times t}\times 100$

Eq. 1

Where* y* stands for the percentage of drug released at time *t*.

*Physicochemical characterisation*

Selected formulations were characterised for the following parameters:°

*Particle size and polydispersity index*

A Malvern Zetasizer NanoZS (Malvern, United Kingdom) was used to measure particle size and polydispersity index (PDI) of BZ NEs without any dilution. Light scattering was monitored at 25 ºC and a 90º angel.

*pH measurement*

The pH of samples was measured by a pH meter (Mettler Toledo, Switzerland) at 25 °C.

*Refractive index*

The refractive index of samples was determined at 25 °C using an Abbe-type refractometer (Shanghai Optical Instrument Factory, China).

*Osmolality*

A Vapour pressure osmometer (Wapro, Wescor Inc., USA) was employed to measure the osmolality of BZ NEs.

*Viscosity*

Rheological measurements were conducted with a Brookfield DVIII viscometer (Brookfield Engineering Laboratories Inc., USA) using spindle No. 40 at 25.0 ± 0.5 °C. The rotation speed was increased up to 150 rpm and the viscosity was determined from the linear portion of the rheogram where the shear stress (dynes/cm^2^) was plotted against the shear rate (s^-1^).

*Accelerated physical stability*

Three different procedures including heating-cooling cycles, freeze-thaw cycles and centrifugation were performed to monitor the physical stability of BZ NEs. After each test, all samples were monitored for phase separation, clarity and any other physical instability. Stable formulations during these studies were subjected to future investigations as described below. 

*Heating-cooling cycles*

Six cycles between 4 and 40 °C, keeping formulations at each temperature for at least 48 h. were performed (25).

*Freeze-thaw cycles*

Three freeze-thaw cycles were performed between -21 and +25 °C with a minimum of 48 h storage of samples at each temperature (26).

*Centrifugation*

Those formulations which passed the previous tests were centrifuged at 13,000 rpm for 30 min (27, 28, 29).

*In-vivo therapeutic studies*

Five New Zealand albino rabbits weighing 1.5-2 kg with normal IOP were used for animal studies. Animal studies were performed according to the approved protocol of Institutional Animal Care and Use Committee of Shahid Beheshti University of Medical Sciences. Rabbits were kept in an air conditioned room with regular light and dark cycles (12 h) at 25 ºC. Single-dose and cross-over design study was performed to evaluate the therapeutic efficacy of BZ NEs in comparison to the marketed BZ suspension. 

Samples (50 μL) were instilled topically into the right eye of rabbits with left eyes serving as controls. At defined time points (30, 60, 120, 180, 240, 300 and 360 min after instillation) the IOP was determined with a rebound tonometer (Icare, Finland). The average of five consecutive IOP readings was recorded and the mean was determined. By calculating the IOP percentage reduction at each time point, three different pharmacodynamic parameters including the maximum percentage decrease in IOP (E_max_), the time required to gain maximum reduction in IOP (T_max_) and the area under the curve of percentage decrease of IOP versus time (AUC_0-6h_) were calculated. 

*Statistical analysis*

All experiment were performed in triplicate and data presented as mean±SD. One-way analysis of variance (ANOVA) with Tukey as a post test was applied to determine significant differences between samples in the release studies. For *in-vivo* therapeutic efficacy studies, an unpaired t-test was performed. P < 0.05 was selected as significant level for both analyses.

## Results and Discussion

*Development of BZ NEs*

Four different non-ionic surfactants (Brij 35, Cremophor( RH40), Labrasol and Tyloxapol), two oils (Triacetin and Capryol 90) and Transcutol-P as co-surfactant were used to construct partial pseudoternary phase diagrams (data not shown). Seven primary BZ NE combinations were then selected. By altering the amount of oil (b series) or S_mix_ (c series) in the primary formulations (a series), 21 nanoemulsion were prepared. Of these formulations two samples (NE5b and NE7b) became milky after adding BZ and were thus excluded from further studies, with the remaining nineteen formulations undergoing further investigations (Table 2).

*In-vitro drug release studies*

**Figure 1:**
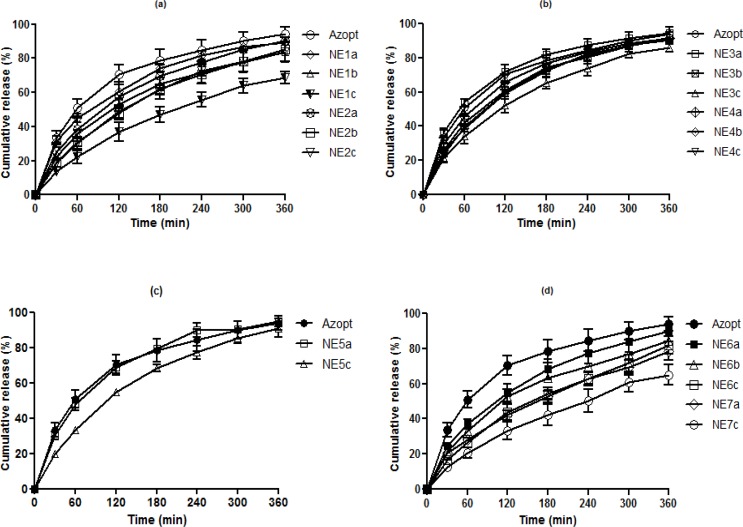
*In-vitro * release profiles of BZ-loaded NEs and Azopt based on different surfactants:

O/W NEs are suitable vehicles for sustained drug delivery by allowing incorporation of lipophilic drugs such as BZ in their internal oily phase. In addition, *in-vitro* release data from such colloidal systems is valuable in predicting their *in-vivo *performance (30), thus the *in-vitro* release studies were used for pre-screening of the prepared BZ NEs before moving into *in-vivo* studies. Based on the release efficiency of different formulations compared to the commercial suspension of BZ (Azopt^®^) at 60 and 360 min, samples with the lowest RE% in each group were selected for physicochemical characterisation, stability and therapeutic efficacy studies. Table 3 shows the REs of all formulations in comparison to Azopt at 60 and 360 min including statistical significance. As shown in Figure 1, all developed formulations (b and c series) exhibited a sustained release pattern compared to Azopt and their respective primary formulations (a series).

NE1c showed the lowest RE% at both time points (12.71 and 43.62% respectively). These results indicate that by increasing the percentage of the internal phase, the release rate of BZ can be reduced. This trend was also observed when the oil in group II was changed to Capryol 90 instead of Triacetin. Also, it seems that the lower RE% of NE1c in comparison with NE2c resulted in higher solubility of BZ in Triacetin (data not shown). Incorporation of more lipophilic drug in the oily core of NE1c led to slower drug release (31). In both groups, developed formulations (NE1b, NE1c, NE2b and NE2c) with significantly lower RE% values in comparison to Azopt were chosen for subsequent studies.

In the groups consisting of Cremophor (RH40), Transcutol P and Triacein or Capryol 90 (Group III and IV), similar to the previous two groups, NE3c with a RE of 20.12 and 58.98% respectively was selected for further investigations, while NE4b and NE4c both showed significantly lower RE% values only at 60 min but were both still selected for subsequent studies. In group V,( NE5c) showed the lowest RE% compared to Azopt and NE5a, thus it was chosen for the therapeutic efficacy experiments.

Results revealed that formulations containing Tyloxapol as a surfactant in all primary and secondary formulations had lower RE values compared to the commercial formulation and two formulations from each group (NE6b, NE6c, NE7a and NE7c) were selected for further investigations, although NE6c and NE7c showed the lowest RE% in their groups. In contrast to groups I and II, substitution of Triacetin by Capryol 90 reduced RE%. This may be attributed to the higher viscosity of (NE7c) in comparison to (NE6c) (Table 4). In summary, based on the release profiles of the different NEs compared to (Azopt) (Figure 1) the following observations could be made:

- In all series c formulations, the percentage of drug released was decreased compared to series a which was attributed to the lower thermodynamic activity of BZ due to an increase in Smix (32).
- Adding oil to the developed formulations (series b) in comparison to primary formulations (series a) resulted in a reduction of RE%. This was in agreement with previous reports (33, 34).
- Addition of S_mix_ (series c) in comparison to nano emulsions with a higher amount of oil (series b) led to a further decrease in RE% in all groups. This can be explained by the higher viscosity of series c formulations (33, 35) and the inhibition of diffusion by the covering of oil with higher surfactant amounts (35).
- All NEs had a lower RE% in comparison to commercial (Azopt). It seems the tendency of BZ to remain in the internal oily phase could result in a low driving force for transport to the external aqueous phase (30).

*Physicochemical characterization*

Based on the *in-vitro* drug release studies twelve formulations, marked with a √, were selected for further physicochemical property evaluations. A summary of the results is presented in Table 4. 

*Particle size analysis and polydispersity index*

One of the most important properties of NE systems is the oil droplet size distribution. The mean droplet size of all prepared NEs was within the nanometer range (7.53 to 48.67 nm). The incorporation of co-surfactants caused more fluidity of the interfacial film and increased the radius curvature of the droplets, thus transparent nanodroplets were obtained (36). 

The results also showed that by increasing the S_mix_ concentration at fixed oil content (5%) from 20% (NE7a) to 30% (NE7c), the globule size decreased (from 16.76 to 10.52 nm). This may be attributed to S_mix_ and its capability to reduce the interfacial tension between oil and water (37).

The ratio of standard deviation to the mean droplet size is expressed as polydispersity index (PDI), as such the ( PDI) indicates the uniformity of the droplet size within the formulation (38, 39). All PDI values were below 0.4 which confirmed a narrow size distribution of oil globules in the formulations.

*PH measurement*

The appropriate pH for topical ophthalmic formulations is between 6.6 to 7.8 (40). However, tears are able to buffer formulations that are slightly outside of this range (13). 

All prepared formulations were within a pH range of 5.89 to 6.56 and were thus found to be in acceptable due to the buffering capacity of the tears.

*Refractive index*

Measuring the refractive index is a way to confirm the isotropic nature of the NEs (12) which is also important to avoid blurred vision (13). Generally, the refractive index of eye drops should be lower than 1.476 to avoid visual interference (41). Low refractive index values (1.367-1.384) were found for all NE formulations which confirmed their isotropic nature.

*Osmolality*

The osmolality of the prepared NEs was in range of 645.3 to 1551 mmol/kg. Although these values are above the commonly acceptable range of 100 to 640 mOsmol/kg (42), formulations with osmolality values up to 2400 mOsmol/kg are generally considered acceptable according to Hasse and Keipert’s study (43).

**Table 1:** Composition of the primary BZ NEs

**Formulation**	**Oil**	**Surfactant**	**Co-surfactant**	**S**_mix_ ** Ratio**
NE1	Triacetin	Brij 35	Transcutol-P	2-1
NE2	Capryol 90	Brij 35	Transcutol-P	2-1
NE3	Triacetin	Cremophor RH40	Transcutol-P	1-1
NE4	Capryol 90	Cremophor RH40	Transcutol-P	1-1
NE5	Triacetin	Labrasol	Transcutol-P	2-1
NE6	Triacetin	Tyloxapol	Transcutol-P	2-1
NE7	Capryol 90	Tyloxapol	Transcutol-P	2-1

**Table 2:** Composition of developed NEs from the primary formulations

**Group**	**Formulation**	**Oil%**	**S**_mix_ **%**	**S**_mix_ ** Ratio**
I	NE1a	5	20	2-1
	NE1b	7.5	20	2-1
	NE1c	5	30	2-1
II	NE2a	5	20	2-1
	NE2b	7.5	20	2-1
	NE2c	5	30	2-1
III	NE3a	5	20	2-1
	NE3b	7.5	20	2-1
	NE3c	5	30	2-1
IV	NE4a	5	20	1-1
	NE4b	7.5	20	1-1
	NE4c	5	30	1-1
V	NE5a	5	20	2-1
	NE5c	5	30	2-1
VI	NE6a	5	20	2-1
	NE6b	7.5	20	2-1
	NE6c	5	30	2-1
VII	NE7a	5	20	2-1
	NE7c	5	30	2-1

**Table 3:** *In-vitro* drug release of BZ from NEs and Azopt after 60 and 360 min

**Formulation**	**RE(%), 60 min**	**RE(%), 360 min**	**Result** *
Azopt	30.43±3.25	71.05±4.83	-
NE1a	28.42±2.40	66.31±2.22	-
NE1b	20.95±1.50**	57.95±3.81*	√
NE1c	12.71±2.13***	43.62±4.00**	√
NE2a	23.50±4.33	62.87±4.79	-
NE2b	18.49±1.94**	56.00±4.80*	√
NE2c	17.30±2.10**	55.95±2.28*	√
NE3a	33.32±1.69	73.43±1.46	-
NE3b	24.48±3.38	65.64±4.37	-
NE3c	20.12±2.98*	58.98±3.59*	√
NE4a	27.23±2.93	68.41±3.20	-
NE4b	23.49±1.01 *	65.02±1.94	√
NE4c	22.15±1.02 *	64.99±0.93	√
NE5a	28.40±2.24	71.48±1.84	-
NE5c	18.74±0.65**	61.30±1.81 *	√
NE6a	23.10±0.64 **	61.80±4.36	-
NE6b	19.71±0.27***	56.87±1.01 **	√
NE6c	15.56±2.05 ***	50.34±3.73 ***	√
NE7a	18.09±2.44 **	49.68±2.95 **	√
NE7c	11.94±1.79 ***	40.11±4.82 ***	√

(mean ± SD, n=3,

* Samples selected for further evaluations (√)

* p-value<0.5,

** p-value<0.01,

*** p-value<0.001)

**Table 4:** Physicochemical properties of BZ NEs (mean ± SD, n=3

**Formulation**	**Droplet size (nm)**	**PDI**	**pH**	**Refractive index**	**Osmolality** **(mmol/kg)**	**Viscosity** **(cP)**
NE1b	31.20±2.27	0.377±0.025	6.08±0.12	1.367±0.001	960.0±14.1	7.04±0.91
NE1c	42.38±1.62	0.356±0.027	5.89±0.17	1.378±0.001	1109.8±5.9	18.94±3.40
NE2b	20.09±1.70	0.341±0.009	6.18±0.13	1.368±0.002	645.3±8.0	14.31±2.43
NE2c	48.67±2.10	0.375±0.015	6.15±0.11	1.379±0.001	1243.8±4.0	22.36±2.74
NE3c	22.16±0.27	0.376±0.014	6.41±0.06	1.378±0.001	1153.2±4.5	17.63±0.89
NE4b	32.55±0.18	0.264±0.004	6.56±0.23	1.367±0.001	928.8±37.9	3.82±0.30
NE4c	30.90±0.42	0.390±0.007	6.43±0.20	1.377±0.001	1551.0±41.0	14.38±2.65
NE5c	25.11±0.24	0.209±0.017	6.18±0.23	1.376±0.001	1510.0±50.1	5.79±0.44
NE6b	7.53±0.05	0.232±0.015	6.24±0.24	1.372±0.001	862.7±6.6	2.74±0.60
NE6c	8.65±0.67	0.292±0.030	6.55±0.06	1.383±0.001	1203.0±24.0	6.65±0.31
NE7a	16.76±0.07	0.239±0.006	6.45±0.15	1.370±0.003	585.2±13.0	17.59±2.43
NE7c	10.52±0.03	0.253±0.007	6.38±0.08	1.384±0.001	1099.3±40.5	23.96±2.52

**Table 5:** Pharmacodynamic parameters after topical administration of the developed BZ NEs and Azopt

**Formulation**	**E**_max_ ** (%)**	**T**_max_ ** (h)**	**AUC**_0-6h_
Azopt	25.09±3.69	1.80±0.45	97.00±7.92
NE1b	28.56±6.88	0.80±0.27**	109.44±21.30
NE1c	22.78±3.24	1.60±0.89	90.52±11.69
NE2b	33.96±6.48*	1.10±0.27 **	120.74±17.59 *
NE2c	35.24±9.09	1.00±0.61 *	114.79±21.97
NE3c	25.93±4.62	1.20±0.45	95.48±15.91
NE4b	34.71±5.33 *	1.50±1.00	121.41±17.76 *
NE4c	30.29±5.06	1.10±0.55	109.46±7.96 *
NE5c	36.01±6.99 *	1.00±0.61 *	125.08±15.14 **
NE6b	28.44±6.54	0.90±0.65 *	106.95±19.81
NE6c	27.99±7.03	2.00±0.71	88.01±10.99
NE7a	29.02±5.67	2.20±0.55	108.95±15.01
NE7c	37.52±4.90 **	0.90±0.22 **	129.60±11.53***

(mean ± SD, n=5,

* p-value<0.5,

** p-value<0.01,

*** p-value<0.001 compared to Azopt).

(a Brij 35 b Cremophor RH40 c Labrasol and d) Tyloxapol (mean ± SD, n=3).

*Viscosity*

Newtonian behaviour with viscosities between 2.74-23.96 cP was observed for all BZ NEs as expected from NEs with a small droplet size (44). All viscosity values were in a suitable range (lower than 25 cP) for ocular drug delivery to avoid any irritation and thus reflex tearing (45, 46). The effect of surfactant content on viscosity was also evaluated. These studies revealed that by increasing the S_mix_ content from 20 to 30% w/w (series c), the viscosity increased. This may be attributed to the higher hydration of the hydrophilic chains of the surfactants that causes strong interactions via hydrogen bonds (47) thus resulting in higher shear stress.

*Accelerated physical stability*

Three different tests were carried out to evaluate the physical stability of selected formulations. All formulations exhibited good physical stability at these conditions. Only NE1b showed cloudiness at lower temperatures (4 °C), but after returning it to room temperature, clarity was recovered rapidly. Similar observations were obtained when formulations were frozen at -21 °C. It seems that the transient instability is caused by coagulation of the internal phase at low temperatures (17) as well as the pressure of ice crystals on the oil globules and adsorbed layers of S_mix _(48). No other physical instabilities such as creaming, cracking and phase separation were observed during these studies. As a result, all twelve formulations were subjected to* in-vivo* therapeutic efficacy evaluations.

*In-vivo therapeutic studies*

Twelve BZ NEs were investigated for their therapeutic efficacy after instillation into normotensive rabbit eyes. Results for E_max_, T_max_ and AUC_0-6h_ of all NEs and the marketed product (Azopt) are denoted in Table 5. Besides NE1c, all NEs showed higher E_max_ values compared to Azopt. NE7c exhibited the maximum reduction in IOP with a significant difference (P<0.01) in comparison to the commercial product. Values of T_max _indicated that most of the NEs had a faster onset of action compared to Azopt possibly due to the already solubilized drug in the oil phase and the higher penetration of the formulations across the corneal tissues. NE1b had the lowest value for T_max _(P<0.01) and thus the fasted onset of action. The ocular bioavailability of drug from the formulations was determined by calculating the AUC_0-6h_. Similar to the other parameters investigated, most NEs had increased bioavailability compared to Azopt. Within all formulations, NE7c showed the maximum value for AUC_0-6h _compared to Azopt (P<0.001).

It should be noted that in all NE formulations, the amount of BZ was fixed at 0.4% w/w which is 60% lower than the concentration of the marketed product (Azopt 1%). Overall, these results indicate that the NEs enhanced the therapeutic outcomes despite reducing the concentration of the active substance highlighting their improved delivery capabilities compared to the commercially available BZ suspension.

*Effect of oil type on therapeutic efficacy*

By comparing different NE formulations (NE1b, NE1c and NE6c with NE2b, NE2c and NE7c respectively), it was observed that the drug bioavailability from Capryol 90 based formulations was higher than from Triacetin based formulations. Capryol 90 is a mixture of propylene glycol mono- and diesters of fatty acids composed predominately of caprylic acid which can significantly increase corneal permeability of drugs by various mechanisms such as affecting cell membranes and the tight junctions (49).

*Effect of surfactant type on therapeutic efficacy.*

Four Triacetin based formulations were evaluated for the effect of surfactant type on therapeutic efficacy. The values of AUC_0-6h _of these formulations were ranked according to the following order: NE5c>NE3c>NE2c>NE6c. This result indicates that the presence of Labrasol lead to higher drug bioavailability compared to other surfactants. This result is in agreement with previous work that introduced Labrasol as a potential penetration enhancer in ophthalmic drug delivery. Labrasol can improve transcorneal penetration by creating micelles in the epithelium layer leading to membrane solubilisation (50). 

Overall, these results have shown that NEs can act as a valuable drug delivery platform for enhanced ocular bioavailability of BZ. The incorporation of penetration enhancers such as surfactants and oils in the structure of NEs leads to higher therapeutic efficacy. In addition to the reasons discussed above, endocytosis of nanosized particles or droplets can be another reason for the bioavailability enhancement of BZ NEs (51) and should thus be further investigated. Finally, further studies will now be performed to confirm the ocular tolerability of these formulations.

## Conclusions

The spontaneous emulsification method was successfully employed to prepare BZ NEs for ophthalmic drug delivery. The selected formulations exhibited a sustained release profile with appropriate physicochemical characteristics. Various components in NE formulations such as oils and surfactants facilitated BZ penetration into the corneal tissue with lower drug concentrations (0.4%) in comparison with the commercial product (1%) still resulting in higher therapeutic efficacy *in-vivo*. These finding suggest that NEs could be a promising delivery platform for enhanced BZ delivery. However, more investigations are needed to optimise the NE formulations for ophthalmic application. 
